# Increased Heart Rate Variability following Elective Percutaneous Coronary Intervention in Patients with Stable Coronary Artery Disease and Preprocedural Anxiety

**DOI:** 10.1155/2019/3696825

**Published:** 2019-12-20

**Authors:** Tessa Oktaramdani, E. Mudjaddid, Muhadi, Hamzah Shatri

**Affiliations:** ^1^Department of Internal Medicine, Faculty of Medicine Universitas Indonesia–Cipto Mangunkusumo Hospital, Jakarta, Indonesia; ^2^Division of Psychosomatic Medicine, Faculty of Medicine Universitas Indonesia–Cipto Mangunkusumo Hospital, Jakarta, Indonesia; ^3^Division of Cardiology, Faculty of Medicine Universitas Indonesia–Cipto Mangunkusumo Hospital, Jakarta, Indonesia

## Abstract

**Background:**

There is a strong association between chronic ischemia and autonomic imbalance. Percutaneous coronary intervention (PCI) may restore autonomic balance in patients with stable coronary artery disease (SCAD), which is characterized by increased heart rate variability (HRV). Anxiety is often found in patients who are going to undergo invasive procedures and has been identified to induce autonomic imbalance. The aim of our study is to identify the impact of preprocedural anxiety on increased HRV following an elective PCI.

**Methods:**

Our study was a pretest and post-test correlation study involving 44 SCAD patients who underwent elective PCI at Cipto Mangunkusumo National Hospital. The HRV was measured before and after PCI. Anxiety symptoms were evaluated using Hospital Anxiety Depression Score (HADS) questionnaires.

**Results:**

We found a higher increase on HRV parameter following the PCI of subjects in the nonanxiety group compared with the anxiety group (median = 9.11 vs. 2.83; *U* = 154.00; *p*=0.043).

**Conclusions:**

Preprocedural anxiety may inhibit HRV increase following PCI procedure.

## 1. Introduction

Autonomic dysfunction may precipitate several cardiac conditions such as arrhythmias, heart failure, and even sudden cardiac death [1, 2]. Analyzing the autonomic function allow us to predict morbidity and mortality of cardiac patients. Autonomic activity can be assessed using a noninvasive method by analyzing heart rate variability (HRV). Reduced HRV is associated with decreased cardiac flexibility and resistance to stress, which increase the risks of cardiac events. Previous studies by Gomes et al. [3]; Abrootan et al. [4]; and Aydinlar et al. [5] revealed that restoring myocardial perfusion using percutaneous coronary intervention (PCI) may improve autonomic balance, which is characterized by increased HRV.

Preprocedural anxiety is common in patients undergoing any invasive medical procedures, including PCI. Study showed that pre-PCI anxiety was detected in almost 70% of patients who underwent the procedure [6]. Anxiety may induce sympathetic hyperactivity as characterized by reduced HRV. The aim of our study was to analyze the impact of preprocedural anxiety on HRV increase following PCI.

## 2. Methods

A pretest and post-test correlation study involving 44 patients with stable coronary artery disease (SCAD) who underwent elective PCI at Integrated Heart Service Unit, Cipto Mangunkusumo National Hospital, Jakarta, was conducted between May and June 2018. Patient selection was carried out using a consecutive method with the following inclusion criteria: patients with age ≥18 years, normal sinus rhythm, and chronic stable angina with significant stenosis (≥50%) based on angiography examination.

Patients with acute myocardial infarction, any need for emergent CABG after PCI, taking antiarrhythmic (except beta-blockers) drugs, antianxiety drugs, and antidepressant drugs, having a history of stroke, those with permanent brain injury, asthma, and excessive alcohol consumption (≥5 glasses/day for 5 days or more in the last 1 month), and patients with Hospital Anxiety Depression Score (HADS) ≥8 on depression subscale were excluded from the study.

Education level was defined as low-intermediate for elementary-high school graduates and high level for college graduates. Occupation was defined as employee (public and private company), professional (business, medical, and law), retired, and unemployed.

Atherosclerosis risk factors were defined as follows: hypertension was defined as taking antihypertensive drugs and diabetes mellitus was defined as taking hypoglycemic agents. Stable angina was carefully obtained through medical history in most patients. Noninvasive stress tests were done in 9 subjects (21%) with no history of PCI. Coronary stenosis was quantified by visual judgment and TIMI flow grade. Out of 44 subjects undergoing PCI, 23 subjects had full revascularization.

Standard 12-lead ECG and short-term HRV (5-minutes) measurement were performed before PCI and the next day after PCI in the morning using a photoplethysmograph analyzer. Both time domain (standard deviation of normal-to-normal intervals/SDNN; root mean square of the successive differences/RMSSD) and frequency domain (total power/TP; low frequency/LF; high frequency/HF; and LF-HF ratio) parameters were measured to be included in the study.

The Hospital Anxiety Depression Score (HADS) questionnaire was used to assess preprocedural anxiety [7, 8]. It contains 14 multiple-choice questions, divided into two subscales: anxiety and depression. Each consists of seven items, and each item has a score ranging from zero to three. Subjects with anxiety score of ≥8 were classified as case.

The research protocol for our study had already been approved by the Medical Research Ethics Committee Faculty of Medicine Universitas Indonesia/Cipto Mangunkusumo Hospital, and eligible subjects had given their written consent before they were included in the study. All data were processed and analyzed using Statistical Package for the Social Sciences (SPSS) program version 20.0 for Windows.

## 3. Results

Our study population was 44 patients, which consisted of 34 (77.3%) men and 10 (22.7%) women with a mean age of 62.2 ± 6.9 years. There were 40 (90.9%) subjects with hypertension; while 21 (47.7%) subjects had diabetes, which is a traditional risk factor of CAD. Both conditions were well-controlled by antihypertension, oral antidiabetic drugs, and insulin. The baseline characteristics of our study population are summarized in Table 1.

Subjects were divided into two categories based on the result of HADS anxiety subscales, which were noncase (0–7) and case (8–21) subjects as can be seen in Table 2. The HRV parameters were measured before and after PCI and presented in Table 3. Increased HRV parameters (SDNN) following PCI in both anxiety and nonanxiety groups were compared and analyzed as presented in Table 4.

## 4. Discussion

The characteristics of study participants regarding gender resemble those explained in previous studies of people with SCAD [9–11], in which male subjects are more frequently exposed to major risk factors such as smoking and hypertension. Out of 44 SCAD subjects undergoing elective PCI, 24 subjects (54.5%) experienced preprocedural anxiety. Our finding is consistent with results of previous studies, which demonstrates that more than half patients undergoing PCI experience anxiety [6, 12, 13].

Although 80% of our subjects had a history of PCI, more than half still experienced anxiety throughout the study. Studies by Trotter et al. [6] and Delewi et al. [13] showed no differences in anxiety prevalence between patients who had previous PCI with those who had PCI for the first time.

We have 21 subjects with diabetes, 11 (52%) from the nonanxiety group and 10 (48%) from the anxiety group. There are also 40 subjects (91%) who had hypertension. Since high blood pressure and poor glycemic control is associated with reduced HRV, all hypertensive and diabetic subjects included in our study must be in optimal blood pressure and glycemic control (blood pressure <140/90 mmHg, fasting plasma glucose <130 mg/dL, and post prandial plasma glucose <180 mg/dL, or HbA1C <7.5%) [14, 15].

Eliminating ischemia through PCI can reduce central sympathetic activity, which may serve as the main source of autonomic imbalance in patients with SCAD. Therefore, coronary revascularization may restore autonomic balance, which is characterized by increased heart rate variability towards an optimal level. Previous studies have reported that SDNN of <50 ms is associated with higher morbidity and mortality of cardiac patients, while patients with RMSSD >20 ms, TP > 1000 ms^2^, and LF/HF ratio of 1.5 to 2 are classified as healthy subjects [16, 17].

SDNN is the standard parameter for medical stratification of cardiac risks. It correlates with both morbidity and mortality of cardiac patients [16, 17] Our study revealed that the increase of SDNN following PCI is significantly higher in subjects without preprocedural anxiety compared with those with anxiety (median = 9.11 vs. 2.83; *U* = 154.00; *p*=0.043). Acute anxiety triggered by acute stress could create a state of sympathetic hyperactivity, which is a condition that might hamper HRV increase even after successful revascularization [18, 19].

The limitation of our study may be the small number of the patients. As a result, designing a larger study with more cases could be more informative. Simple interventions such as psychoeducation and psychopharmacology may relieve anxiety and also prevent acute anxiety from becoming a chronic long-standing condition. Harkness et al. [20] explained that psychoeducation can improve the quality of life, angina symptoms, satisfaction level, and disease perception of SCAD patients waiting for elective catheterization. It remains to be determined whether the approach can optimize cardiac autonomic improvement following PCI.

## 5. Conclusion

Preprocedural anxiety may inhibit optimal HRV increase in SCAD patients following elective PCI. Treatment studies should be carried out to determine the impact of anxiety treatment on post-PCI HRV improvement.

## Figures and Tables

**Table 1:** Subject characteristics.

Characteristics	*n* = 44
Age, means (SD)	62.2 (6.9)

Sex, *n* (%)	
Male	34 (77.3)
Female	10 (22.7)

Education level, *n* (%)	
Low-intermediate	19 (43.2)
High	25 (56.8)

Occupation, *n* (%)	
Employee	6 (13.6)
Professional	20 (45.5)
Retired	6 (13.6)
Unemployed	12 (27.3)

History of revascularization (PCI), *n* (%)	
Yes	35 (79.5)
No	9 (20.5)

Coronary artery involvement, *n* (%)	
Single vessel disease	4 (9.1)
Multiple vessel disease	40 (90.9)

Diabetes, *n* (%)	21 (47.7)

Hypertension, *n* (%)	40 (90.9)

Smoking in the last 1 year, *n* (%)	5 (11.4)

**Table 2:** Proportion of anxiety in patients undergoing PCI.

	*n* (%)
Nonanxiety	20 (45.5)
Anxiety	24 (54.5)

**Table 3:** Comparison of HRV before and after PCI.

Parameters	Nonanxiety (*n* = 20)		*p* value	Anxiety (*n* = 24)		*p* value
	Pre-PCI	Post-PCI		Pre-PCI	Post-PCI	
SDNN (ms), median (min-max)	26.19 (11.07–45.46)	39.60 (17.48–103.98)	<0.001	27.19 (15.87–43.16)	28.98 (12.80–59.91)	0.424
RMSSD (ms), median (min-max)	21.90 (10.02–52.40)	30.99 (10.29–59.99)	0.012	29.43 (10.81–91.96)	20.96 (8.82–135.96)	0.753
TP (ms^2^), median (min-max)	523.61 (178.9–1301.35)	550.05 (95.47–15092.0)	0.232	492.15 (208.1–1850.71)	594.64 (105.33–3904.3)	0.607
LF (ms^2^), median (min-max)	107.21 (3.66–1011.58)	101.98 (3.31–1357.97)	0.391	83.71 (6.35–1084.33)	134.02 (7.93–512.83)	0.797
HF (ms^2^), median (min-max)	93.91 (3.57–366.56)	130.64 (6.63–428.78)	0.167	155.40 (6.81–998.93)	83.27 (5.92–1533.92)	0.648
LF/HF, median (min-max)	1.35 (0.27–8.76)	1.62 (0.16–9.31)	0.940	1.07 (0.09–7.88)	1.28 (0.15–6.02)	0.067

SDNN = standard deviation of all normal-normal intervals; RMSSD = root mean square of the successive differences; TP = total power; LF = low frequency; HF = high frequency; LF/HF = ratio LF : HF.

**Table 4:** Increased SDNN following elective PCI.

SDNN increase (pre-post PCI)	Nonanxiety (*n* = 20)	Anxiety (*n* = 24)	*p* value
∆ SDNN median (min-max)	9.11 (−5.19–70.40)	2.83 (−18.90–33.19)	0.043

## Data Availability

The HRV data used to support the findings of this study are available from the corresponding author upon request.
